# Structural, Optical and Photocatalytic Activity of Multi-heterojunction Bi_2_O_3_/Bi_2_O_2_CO_3_/(BiO)_4_CO_3_(OH)_2_ Nanoflakes Synthesized via Submerged DC Electrical Discharge in Urea Solution

**DOI:** 10.1186/s11671-022-03714-3

**Published:** 2022-08-17

**Authors:** E. Hashemi, R. Poursalehi, H. Delavari

**Affiliations:** Department of Materials Engineering, Tarbiat Modares University, Tehran, 14115-143 Iran

**Keywords:** Bi_2_O_3_/Bi_2_O_2_CO_3_/(BiO)_4_CO_3_(OH)_2_ nanoflakes, Multi-heterojunction, Photocatalyst, Optical properties, Submerged DC electrical discharge, Urea solution

## Abstract

In this research, a novel ternary multi-heterojunction Bi_2_O_3_/Bi_2_O_2_CO_3_/(BiO)_4_CO_3_(OH)_2_ photocatalyst is fabricated via submerged DC electrical arc discharge in urea solution. FT-IR, XRD, EDS and PL results confirm the formation of Bi_2_O_3_/Bi_2_O_2_CO_3_/(BiO)_4_CO_3_(OH)_2_ multi-heterojunction. Formation of nanoflake morphology is revealed by FE-SEM and TEM images. The optical properties and intense absorption edge of Bi_2_O_3_/Bi_2_O_2_CO_3_/(BiO)_4_CO_3_(OH)_2_ reveal the proper visible light absorbing ability. The photocatalytic performance of the sample is investigated via the degradation of methylene orange (MeO) and rhodamine B (RB) under visible light irradiation. The photocatalytic activity of Bi_2_O_3_/Bi_2_O_2_CO_3_/(BiO)_4_CO_3_(OH)_2_ is compared with the synthesized sample in water, Bi_2_O_3_/Bi/Bi(OH)_3,_ which exhibits much higher photocatalytic activity. Also, the stable photodegradation efficiency of Bi_2_O_3_/Bi_2_O_2_CO_3_/(BiO)_4_CO_3_(OH)_2_ after four cycles reveals the long-term stability and reusability of the synthesized photocatalyst. The PL intensity of Bi_2_O_3_/Bi_2_O_2_CO_3_/(BiO)_4_CO_3_(OH)_2_ shows an improved separation rate of electron–hole pairs and so enhanced photocatalytic performance. The improved photocatalytic activity can be ascribed to the formation of multi-heterojunctions, flake morphology and intrinsic internal electric field (IEF). Multi-heterojunction nanoflakes enhance the absorbance of visible light and facilitate the separation and transport of photogenerated electron holes through large IEF. Our work offers an effective method for the production of innovative bismuth-based photocatalyst with excellent prospects for the degradation of environmental pollutants and light harvesting for renewable energy generation under visible light.

## Introduction

Energy crisis and environmental pollution are one of the major global problems in the present century [1]. Hence, semiconductor photocatalysts have attracted great attention in this matter. Photocatalysis with solar energy is one of the most advanced and encouraging techniques in reducing energy crisis and environmental pollution [1–, 2, 3, 4]. The light of the sun as an inexhaustible energy source is used for the removal of environmental pollutants as well as water splitting and CO_2_ photoreduction to generate hydrogen and carbon-containing fuels to overcome energy scarcity [5–, 6, 7, 8]. So, the synthesis of efficient photocatalysts with high performance in absorbing visible light and charge separation as well as photochemical stability is a key challenge [9]. For example, three-dimensional hierarchical CuCo_2_S_4_ microspheres with considerable photocatalytic activity in removing tetracycline hydrochloride. The high photocatalytic activity is related to the high charge separation efficiency as well as the large specific surface area [10]. Visible-light-driven photocatalysts with heterojunctions have found much attention in the degradation of a wide range of toxic compounds, especially hazardous dyes in wastewater [11, 12]. The photocatalytic activity depends not only on the material but also on the dimensions and structure as well as the composition of the photocatalysts [13, 14]. Bismuth-based complex oxide semiconductors are new promising materials for environmental remediation, water splitting and photocatalytic applications, which show a good photocatalytic performance under visible light [15].Among the diverse photocatalytic materials such as TiO_2_, ZnO, SnO_2_, WO_3_ and CeO_2_, bismuth-based photocatalysts are an important group with low toxicity compared to other photocatalysts which have photocatalytic performance for the degradation of organic pollutants in the environment as well as industrial hazardous wastes [16, 17]. In the electronic structure of bismuth oxide, the valence band consists of O 2*p* and Bi 6*s* hybrid orbitals. The Bi 6*s* orbital raises the mobility of photogenerated charge carriers and thus leads to a decrease in the band gap [15, 18]. Bi_2_O_3_ is a p-type semiconductor which has a direct band gap energy of less than 3.0 eV [19]. There are six main polymorphs for Bi_2_O_3_, which crystallize into different crystal structures [20]. The monoclinic α-Bi_2_O_3_ phase is the most stable phase at room temperature [21]. Thermodynamically, α-Bi_2_O_3_ is stable between 25 and 730 °C and then transformed to face-centered cubic δ-Bi_2_O_3_ phase at 730 °C and remains stable up to its melting point at 825 °C [20, 22]. The tetragonal β-Bi_2_O_3_ and body-centered cubic γ-Bi_2_O_3_ are metastable phases, which are formed at high temperatures and can stabilize down to room temperature or transform to α-Bi_2_O_3_ at high cooling rates [23, 24]. In addition, thermal transformation of α-Bi_2_O_3_ and β-Bi_2_O_3_ to Bi_2_O_2_CO_3_ and (BiO)_4_CO_3_(OH)_2_ was reported previously [25, 26]. Bismuth oxides, including α-Bi_2_O_3_, β-Bi_2_O_3_, γ-Bi_2_O_3_ and δ-Bi_2_O_3_, as well as Bi_2_O_2_CO_3_ and (BiO)_4_CO_3_(OH)_2_ show photocatalytic activity [20, 23–, 26, 27, 28, 29]. However, the small specific surface area, chemical instability and high recombination rate of photogenerated carriers of Bi_2_O_3_ demonstrate the main problem for applications of Bi_2_O_3_ as a photocatalyst merely [15]. Several approaches are developed to overcome the drawbacks of using a single-phase semiconductor as a photocatalyst, namely metal or ion doping, establishing composites or formation of multi-junctions [21–, 30, 31, 32]. Among them, the formation of multi-junctions has gained a lot of attention and has been used widely [32]. The construction of a heterojunction can efficiently improve photocatalytic activity by increasing the separation of photogenerated electrons and holes effectively, for example, 0D/2D S-scheme N-CDs/S-C_3_N_4_ heterojunction with excellent photocatalytic activity in degradation of organic pollutants and H_2_ evolution. The improved photocatalytic performance was attributed to the formation of S-scheme heterojunction with high redox potentials between nitrogen-doped carbon dots (N-CDs) and sulfur-doped carbon nitride (S-C_3_N_4_) semiconductor [33]. In addition, multi-heterojunctions can show better charge transport mechanism and thus higher photocatalytic activity. As an example of a heterogeneous photocatalytic reaction with efficient carrier separation and well-matched energy band structure, can refer to the organic/inorganic PDI-Urea/BiOBr S-scheme heterojunction with photocatalytic performance for the degradation of antibiotics such as ofloxacin, tetracycline as well as high–low junction OV-BiOBr/Cu_2−*x*_S with double defects namely, oxygen and copper vacancy for hydrogen evolution [34, 35]. In addition to these, we can refer to visible light photo-response AgI-loaded BiOI, Bi_2_S_3_/Bi_2_O_3_/Bi_2_O_2_CO_3_ ternary nanocomposites, Z-scheme Ag–AgI/BiOI–Bi_2_O_3_ photocatalysts and SnO_2_/Bi_2_O_3_/BiOI nanofibers with enhanced photocatalytic performance [36–, 37, 38, 39]. IEF is a factor in improving photocatalytic activity by means of simplifying transformation and separation of the photogenerated carriers. The alternative layer structure of (Bi_2_O_2_)^2+^ fluorite flake and (MO_6_)^2−^ perovskite layers in bismuth-based binary metal oxides form IEF that enhanced the separation efficiency of photogenerated charge carriers [26, 40]. Bismuth subcarbonate with Bi_2_O_2_CO_3_ chemical formula is an n-type photocatalyst and has shown photocatalytic performance in the decomposition of pollutants in wastewater and polluted air [15, 41, 42]. The electronic structure of Bi_2_O_2_CO_3_ is composed of hybridized O 2*p* and Bi 6*p* orbitals in the conduction band and O 2*p*, Bi 6*p* and C 2*p* orbitals in the valence band [15]. The structure of Bi_2_O_2_CO_3_ is composed of (Bi_2_O_2_)^2+^ slabs that are intertwined by means of CO_3_^2−^ layers, which lead to a large IEF and improved photocatalytic performance [26, 43]. However, the photocatalytic application of Bi_2_O_2_CO_3_ under the solar spectrum is limited due to the wide band gap between 2.8 and 3.5 eV and low charge separation efficiency [26, 43, 44]. Hence, the formation of junctions between Bi_2_O_2_CO_3_ and other visible responsive photocatalytic semiconductors like α-Bi_2_O_3_ is an appropriate way to extend the photocatalytic activity even under visible light, such as α-Bi_2_O_3_/Bi_2_O_2_CO_3_ and Bi_2_O_2_CO_3_/Bi_2_S_3_, Bi_2_O_2_CO_3_/BiOCl [44, 45, 46]. (BiO)_4_CO_3_(OH)_2_ as an Aurivillius oxide with 2.17 eV band gap is a promising visible-light driven photocatalyst [26]. (BiO)_4_CO_3_(OH)_2_ has the same structure as Bi_2_O_2_CO_3_ and can be formed by replacing CO_3_^2−^ layers of Bi_2_O_2_CO_3_ with OH^−^ layers which are more polar and increase IEF of photocatalyst and thus lead to more photocatalytic performance [26, 47]. Therefore, Bi_2_O_2_CO_3_ and (BiO)_4_CO_3_(OH)_2_ are suitable candidates for construction of layered nanostructures with a large IEF and a high ability to separate photogenerated carriers [26, 43]. Consequently, the formation of junctions between (BiO)_4_CO_3_(OH)_2_ and Bi_2_O_2_CO_3_ can improve visible light photocatalysis activity [26, 48]. The interfaces between (BiO)_4_CO_3_(OH)_2_ and Bi_2_O_2_CO_3_, which are nearly contacted and have an efficient built-in electric field, improve the separation of photogenerated carriers and lead to better photocatalytic performance. (BiO)_4_CO_3_(OH)_2_/Bi_2_O_2_CO_3_ with internal polarized heterojunction was synthesized and shows enhanced photocatalytic activity due to the synergetic effect between IEF and carriers separation and indicated great potential in the application for wastewater treatment [26]. There are many growing numbers of methods for the preparation of single-phase or multi-heterojunctions of bismuth-oxide-based nanostructures. Hydrothermal synthesis of α-Bi_2_O_3_ photocatalysts with nanosheet structure, Bi/Bi_2_O_3_ nanoparticles’ synthesis by Nd:YAG laser ablation, solvothermal preparation of Bi/Bi_2_O_2_CO_3_ heterojunction photocatalyst, synthesis of photocatalytic dendritic α-Bi_2_O_3_/Bi_2_O_2_CO_3_ heterostructures with facile transformation, two-dimensional (BiO)_4_CO_3_(OH)_2_/Bi_2_O_2_CO_3_ heterostructure photocatalyst formation by in situ photosynthesis and flowerlike Au/Bi_2_O_2_CO_3_/Bi_2_O_3_ multi-heterojunction photocatalysts preparation by one-pot in situ growth, which are reported elsewhere [26, 43–, 49, 50, 51, 52]. Compared with several conventional techniques for nanostructures synthesis, DC electrical arc discharge in liquid provides a straightforward, flexible and cost-effective method for mass production of nanomaterials without environmental footprints [53, 54]. There are some experimental parameters including electrical current, composition of electrodes and the chemical nature of the liquid environment, which impress arc discharge process, as well as nanomaterials formation both during and after synthesis in liquid [55, 56]. The chemical nature of the liquid environment is a determining factor in the final composition of synthesized nanomaterials through electrical arc discharge, for instance, TiC nanoparticles synthesis via electrical arc discharge in organic liquids [57, 58]. Furthermore, Bi/Bi_2_O_3_ rodlike nanostructures were prepared via oriented aggregation of bismuth-based nanoparticles synthesized by DC arc discharge in water, where water acts as both a synthesis medium and oxidative environment [53].

In this research, novel Bi_2_O_3_/Bi_2_O_2_CO_3_/(BiO)_4_CO_3_(OH)_2_ multi-heterojunction nanoflakes with considerable photocatalytic performance have been synthesized via DC electrical arc discharge in urea solution for the first time. By applying an appropriate DC current between bismuth electrodes, electrical arc discharge causes plasma generation in the solution and forms Bi_2_O_3_/Bi_2_O_2_CO_3_/(BiO)_4_CO_3_(OH)_2_ nanoflakes. This compound exhibits enhanced photocatalytic performance towards degradations of MeO when compared with both single phases α-Bi_2_O_3_, Bi_2_O_2_CO_3_ and (BiO)_4_CO_3_(OH)_2_ compounds based on previous studies and synthesized Bi_2_O_3_/Bi/Bi(OH)_3_ by arc discharge in water. The photoactivity enhancement can be attributed to the formation of multi-heterojunction nanoflake morphology and improved IEF in Bi_2_O_3_/Bi_2_O_2_CO_3_/(BiO)_4_CO_3_(OH)_2_ nanostructure. To the best of our knowledge, no research has been conducted around the investigation of photocatalytic performance of synthesized ternary Bi_2_O_3_/Bi_2_O_2_CO_3_/(BiO)_4_CO_3_(OH)_2_ nanocomposite by electrical arc discharge in liquid with an efficient photocatalytic activity under visible light for energy and environmental application.

## Experimental

Multi-heterojunction Bi_2_O_3_/Bi_2_O_2_CO_3_/(BiO)_4_CO_3_(OH)_2_ and Bi_2_O_3_/Bi/Bi(OH)_3_ nanoflakes were synthesized by facile DC electrical arc discharge method in liquid [53, 54]. The synthesis part of nanostructures comprises of a heat-resistant glass container filled with urea in water solution with a concentration of 10 g/L for the synthesis of Bi_2_O_3_/Bi_2_O_2_CO_3_/(BiO)_4_CO_3_(OH)_2_ and deionized water for the synthesis of Bi_2_O_3_/Bi/Bi(OH)_3_. The urea was Merck with more than 99% purity. Two 99.9% purity bismuth electrodes were placed in front of each other in vertical mode: One electrode was held fixed, while the other one was movable to make electrodes close to each other in both experiments. The diameter and length of both electrodes were about 5 mm and 30 mm, respectively. The synthesis parts were connected to ARC 200 DC power supply, and a 40A DC current was applied to synthesis of nanostructures. The synthesis process begins by bringing electrodes close together. When electrodes were brought into contact, electrical arc discharge was generated and nanostructures formed. Each discharge lasted about 1–2 s and emitted a very shiny blue light. By establishing electrical discharge, the surface of electrodes was eroded through evaporation and sputtering, and then bismuth atoms and clusters were formed and dispersed in the solution at so lower temperature than the arc hot zone [54, 58, 59]. The suspension of dark nanostructures was  formed through synthesis, and the color of suspension became lighter as time passed due to the oxidation of nanostructures.

FTIR spectroscopy was used to determine the functional groups on the surface of the samples. FTIR spectra were conducted by a PerkinElmer spectrometer on samples using standard KBr pellets. To investigate the crystallographic and phase characteristics of the samples, the powder specimen was analyzed by X'Pert MPD system with Co Kα radiation at 1.78897 Å wavelength. To identify the microstructure, chemical composition, morphology and size distribution of nanostructures, a MIRA TESCAN field emission scanning electron microscope (FE-SEM) with energy-dispersive X-ray spectroscopy (EDS) and elemental mapping of nanostructures as well as a Zeiss EM900 transmission electron microscope (TEM) were used at room temperature. The optical properties of colloidal samples were determined by SCO-TECH SP UV-26 UV–visible spectrophotometer at wavelengths between 190 and 1100 nm at different times. To investigate the photocatalytic activity of synthesized samples, the powder specimen was carried out from solution by using a KTS 5000 rpm centrifuge. PL spectra were recorded by an AvaSpec-2048Tech spectrofluorophotometer, where a Nd:YAG laser with 3rd harmonic crystal was used for excitation of the samples at a wavelength of 355 nm.

The MeO and RB were used as the model pollutants to investigate the photocatalytic activity. The photocatalytic activity was done through dispersing of 45 mg photocatalyst powder in an 80 mL solution of dye (2.5 mg/L) in a glass reactor. It should be noticed that the solution was stirred in darkness for about 30 min to establish adsorption–desorption equilibrium between dye molecules and the surface of photocatalysts. Then, the solution was under irradiation for about 180 min while stirring constantly during irradiation to make a uniform mixture. The photocatalytic tests were applied at room temperature, and the setup was held in a chamber under a 15 W visible LED. During the photocatalytic experiment, each 15 min 3 mL of the solution was taken out to measure optical transmission spectra to reveal the concentration of dye by determining the maximum absorption wavelength of dyes. To evaluate the reusability of synthesized sample, cyclic photodegradation experiments of MeO on Bi_2_O_3_/Bi_2_O_2_CO_3_/(BiO)_4_CO_3_(OH)_2_ nanocomposite were done under the same conditions. For doing cyclic photocatalysis tests, Bi_2_O_3_/Bi_2_O_2_CO_3_/(BiO)_4_CO_3_(OH)_2_ photocatalyst was withdrawn from the solution by means of the centrifuge after each cycle. The solution sample is centrifuged at 4500 RPM for 10 min, then washed and cleaned via ultra-sonicating of the sample in deionized water several times and, after drying, reused again in a new solution of MeO with photocatalyst. The photodegradation procedure of MeO by the sample was redone in four cycles. Furthermore, various scavengers were used to investigate the role of reactive oxidative species in the MeO degradation process by Bi_2_O_3_/Bi_2_O_2_CO_3_/(BiO)_4_CO_3_(OH)_2_. To capture the generated holes, hydroxyl and superoxide radicals, the ethylenediaminetetraacetic acid (EDTA), isopropyl alcohol (IPA) and benzoquinone (BQ) were used in the photocatalytic process, respectively.

## Results and discussion

**Fig. 1:**
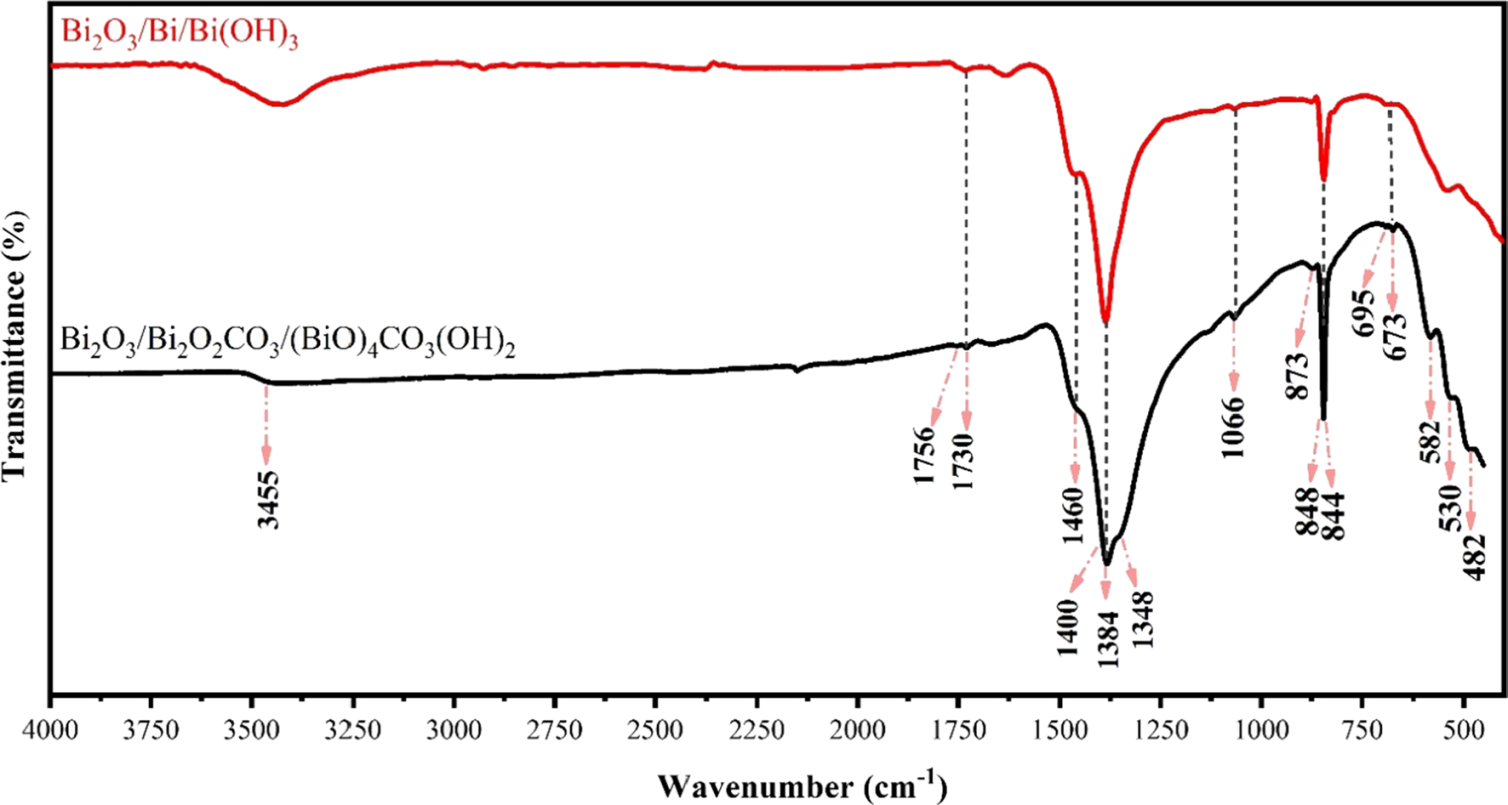
FT-IR spectra of synthesized nanostructures by electrical arc discharge

The type of functional groups on the surface of nanostructures was identified by FTIR spectra, as illustrated in Fig. 1. The FTIR spectra of synthesized samples in urea solution and water are compared. The Bi_2_O_3_/Bi_2_O_2_CO_3_/(BiO)_4_CO_3_(OH)_2_ spectrum reveals characteristic vibrating modes of the free carbonate ion with point group symmetry *D*_3h_. This is engrossed in several internal vibrations, including symmetric stretching mode *ν*_1_ at 1066 cm^−1^, out-of-plane bending mode *ν*_2_ at 848 cm^−1^, antisymmetric vibration *ν*_3_ at 1460 and 1384 cm^−1^, in-plane deformation *ν*_4_ at 695 and 673 cm^−1^ and symmetric stretching plus in-plane deformation modes, *ν*_1_ + *ν*_4_, at 1756 and 1730 cm^−1^[41, 42, 47]. The band groups of CO_3_^−2^ at *ν*_1_, *ν*_2_, *ν*_3_, *ν*_4_ and *ν*_1_ + *ν*_4_ reveal the formation of Bi_2_O_2_CO_3_. Moreover, the bands at 844 cm^−1^ and 300–800 cm^−1^ correspond to Bi-O-C and stretching modes of the Bi-O bonds in Bi_2_O_2_CO_3,_ and Bi_2_O_3_, respectively [60]. The broad bands at 3600–3300 cm^−1^ and 1400 cm^−1^ are corresponding to O–H stretching vibration and O–H bending vibration, respectively, suggesting the presence of (BiO)_4_CO_3_(OH)_2_ in composition [61, 62].

**Fig. 2:**
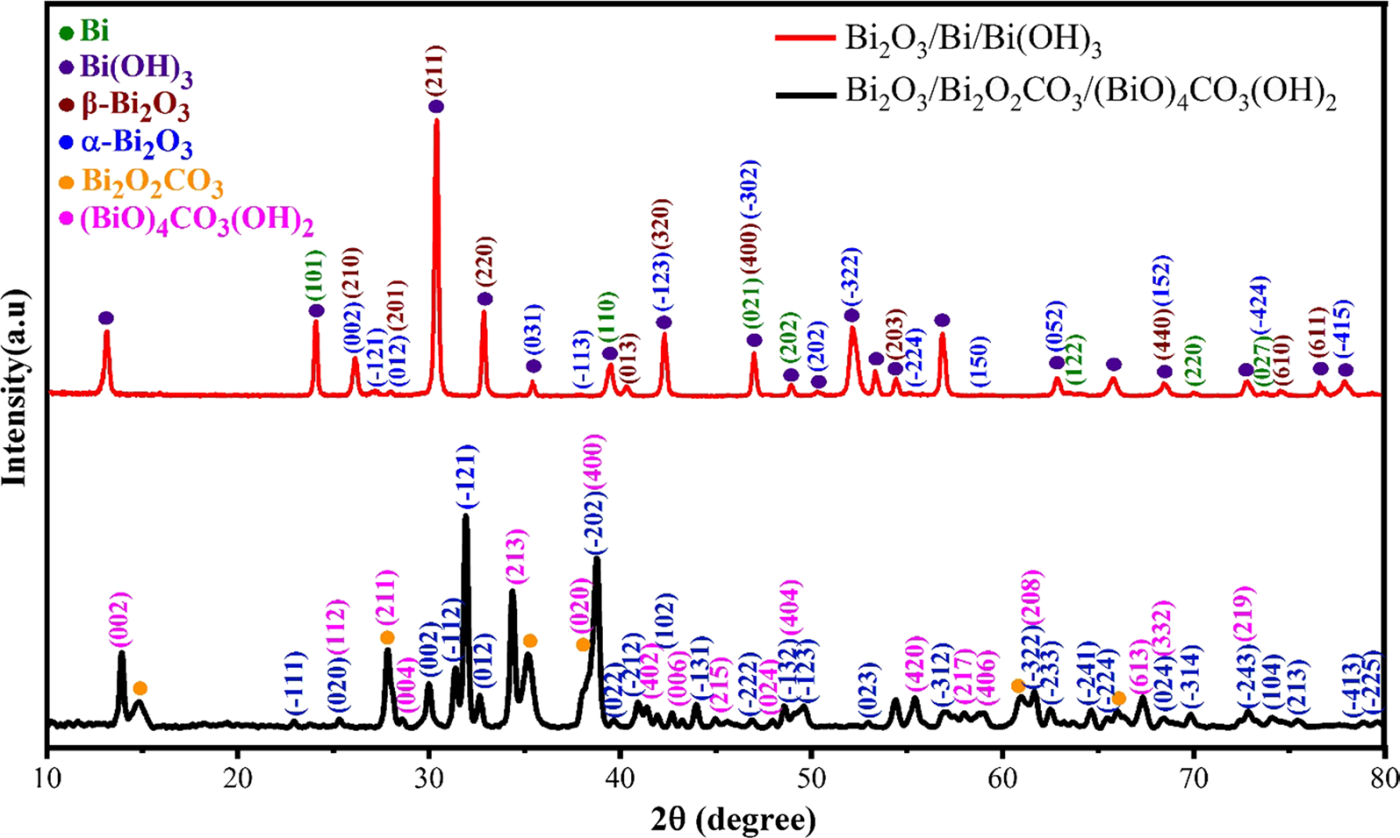
XRD patterns of synthesized nanostructures by electrical arc discharge

The XRD patterns of synthesized samples are illustrated in Fig. 2. It is observed that there are different phases in the pattern of synthesized sample in water, which are related to the crystallographic characteristics of the Bi, Bi_2_O_3_ and Bi(OH)_3_ phases. The diffraction peaks assigned to Bi, α-Bi_2_O_3_, β-Bi_2_O_3_ and Bi(OH)_3_ are based on JCPDS NO. 00-005-0519, 01-071-2274, 00-027-0050 and 00-001-0898 standard cards. The crystallographic parameters of the bismuth phase are rhombohedral, *a* = *b* = 4.5460 Å, *c* = 11.86 Å, *α* = *β* = 90° and *γ* = 120°. Bismuth oxide formed in two crystal systems, monoclinic with *a* = 5.8486, *b* = 8.1661 and *c* = 7.5097 Å, *α* = *γ* = 90°, *β* = 113° and tetragonal with *a* = *b* = 7.7420, *c* = 5.6310 Å, *α* = *γ* = 90° and *β* = 113. In comparison with the pattern of synthesized sample in water, the pattern of the synthesized sample in urea solution reveals α-Bi_2_O_3_, (BiO)_4_CO_3_(OH)_2_ and Bi_2_O_2_CO_3_ phases. The α-Bi_2_O_3_ has the same reference code as that of synthesized in water [63]. The (BiO)_4_CO_3_(OH)_2_ phase is relevant to 00-038-0579 reference code with orthorhombic crystal system and *a* = 10.7716, *b* = 5.4898, *c* = 14.75740 Å and *α* = *β* = *γ* = 90° crystallographic parameters [64]. The crystal structure of Bi_2_O_2_CO_3_ is orthorhombic with a = 5.4680, b = 27.3200, c = 5.4680 Å, *α* = *β* = *γ* = 90° crystal parameters and is based on 01-084-1752 standard card [65]. Based on the calculation of semiquantitative analysis of multiphase systems, the α-Bi_2_O_3_, (BiO)_4_CO_3_(OH)_2_ and Bi_2_O_2_CO_3_ phases have 44%, 36% and 20% shares of structure, respectively. To describe the mechanism of multiphase formation of nanostructures, it should be noticed that establishing electrical arc discharge leads to the formation of a high-temperature plasma [59]. The plasma dissipates the solution molecules and produces neutral, ionic and molecular species of bismuth, water and urea. Formation of Bi, O, C and H ions or OH, H_2_, O_2_, CO and CO_2_ molecules in their ground or excited state in the plasma of electric arc discharge has been reported before [53, 57]. So, based on these species, the formation of oxide, oxide carbonate, hydroxide and carbonate oxide of bismuth could be explained.

**Fig. 3:**
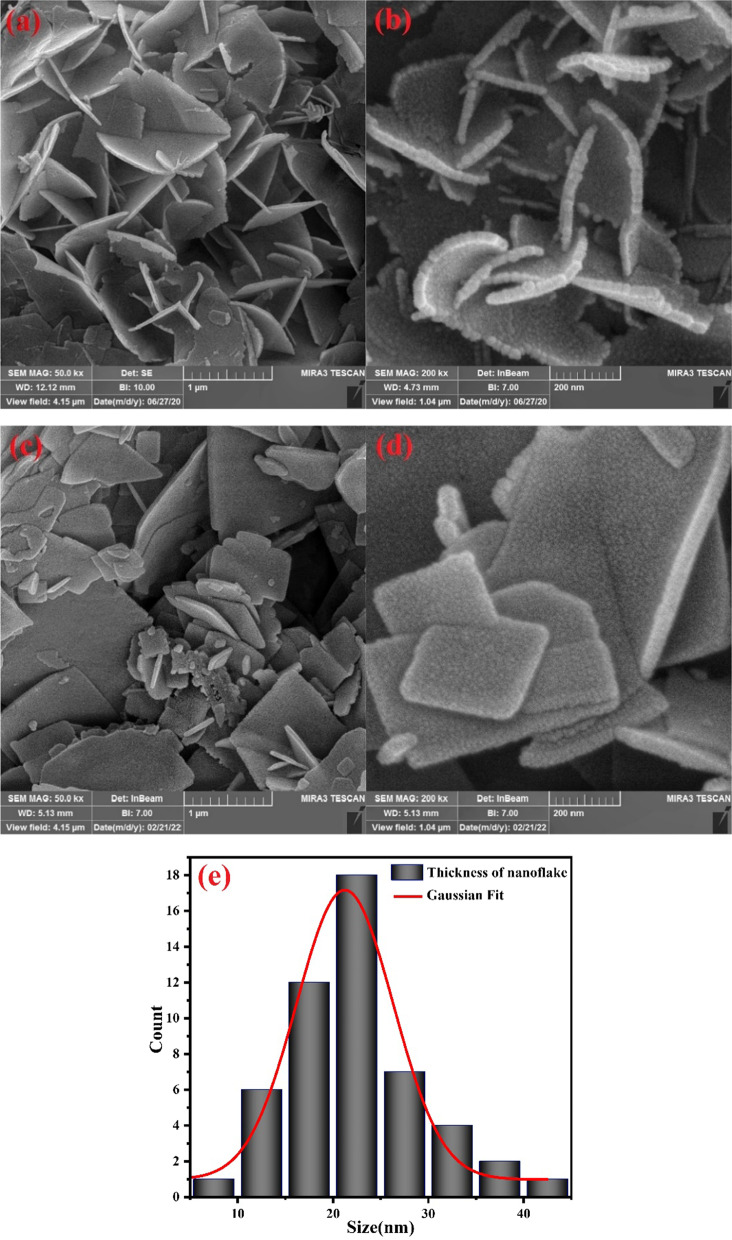
FE-SEM images of synthesized nanostructures by electrical arc discharge in **a**, **b** urea solution, **c**, **d** water, and **e** size distribution histogram of Bi_2_O_3_/Bi_2_O_2_CO_3_/(BiO)_4_CO_3_(OH)_2_ nanoflakes

**Fig. 4:**
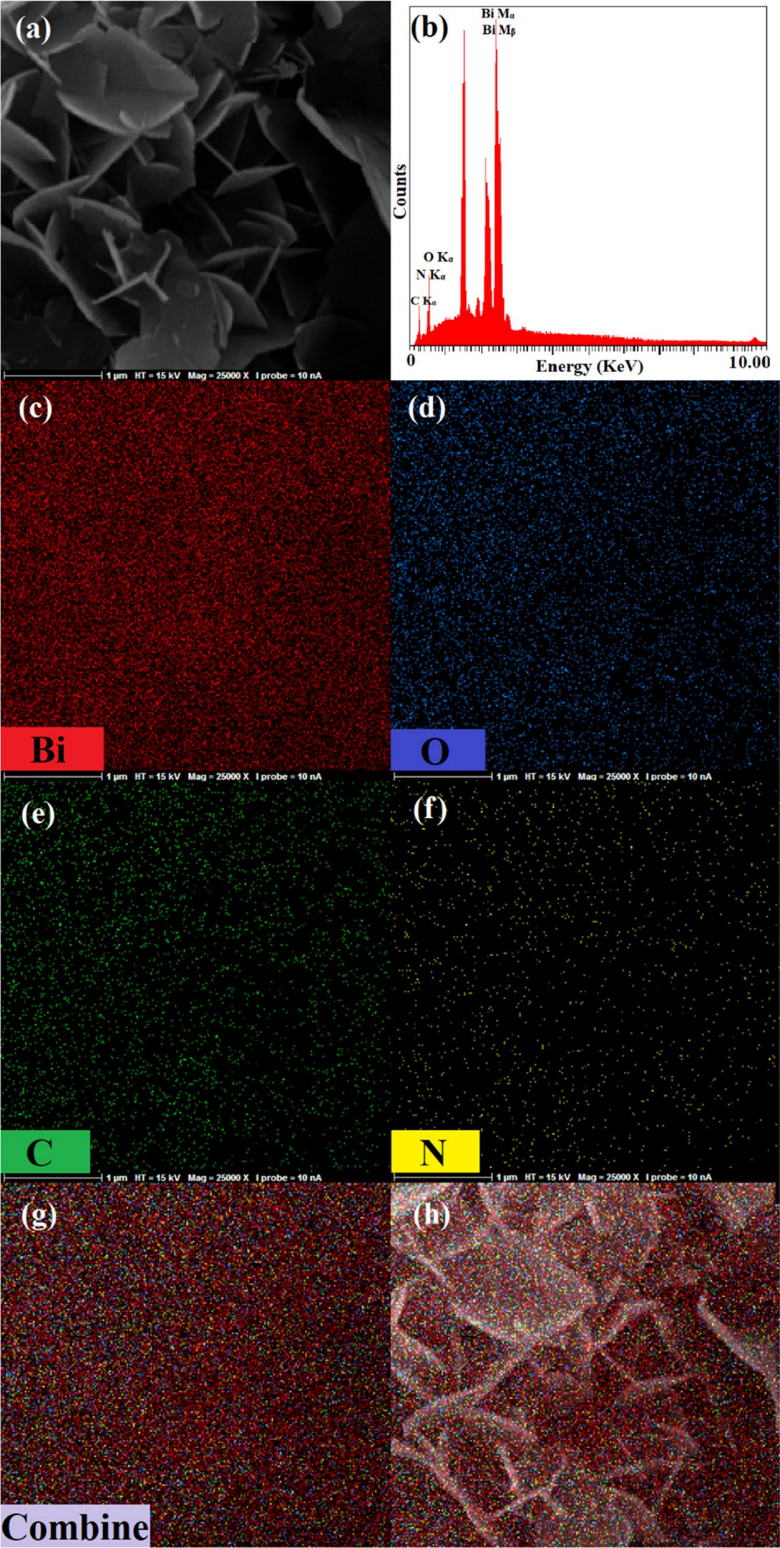
**a** FE-SEM image, **b** EDS spectrum, elemental mapping patterns of **c** Bi, **d** O, **e** C, **f** N, **g** combine and **h** combine with FE-SEM image of Bi_2_O_3_/Bi_2_O_2_CO_3_/(BiO)_4_CO_3_(OH)_2_ nanoflakes

Figure 3 shows the FE-SEM images of samples with different magnifications and size distribution histogram of the synthesized sample in urea solution. The FE-SEM images were obtained after keeping samples in their solutions for 15 days. The Bi_2_O_3_/Bi_2_O_2_CO_3_/(BiO)_4_CO_3_(OH)_2_ has a nanoflake structure, and the approximate average thickness of nanoflakes was demonstrated by a size distribution histogram which was obtained by applying a Gaussian curve on the size histogram. The average thickness of Bi_2_O_3_/Bi_2_O_2_CO_3_/(BiO)_4_CO_3_(OH)_2_ nanoflakes is almost 22.0 ± 0.5 nm. The synthesized sample in water shows a nanoflake structure as well. Even though both samples indicate flake morphology, the Bi_2_O_3_/Bi/Bi(OH)_3_ is considerably more agglomerated than Bi_2_O_3_/Bi_2_O_2_CO_3_/(BiO)_4_CO_3_(OH)_2_ nanocomposite. By increasing the agglomeration of nanostructures, the specific surface area will be reduced which will lead to less photocatalytic performance. Furthermore, FE-SEM elemental mapping and energy-dispersive spectrometry of synthesized nanoflakes in urea solution indicated the uniform distribution of Bi, O and C elements in the structure, as shown in Fig. 4.

**Fig. 5:**
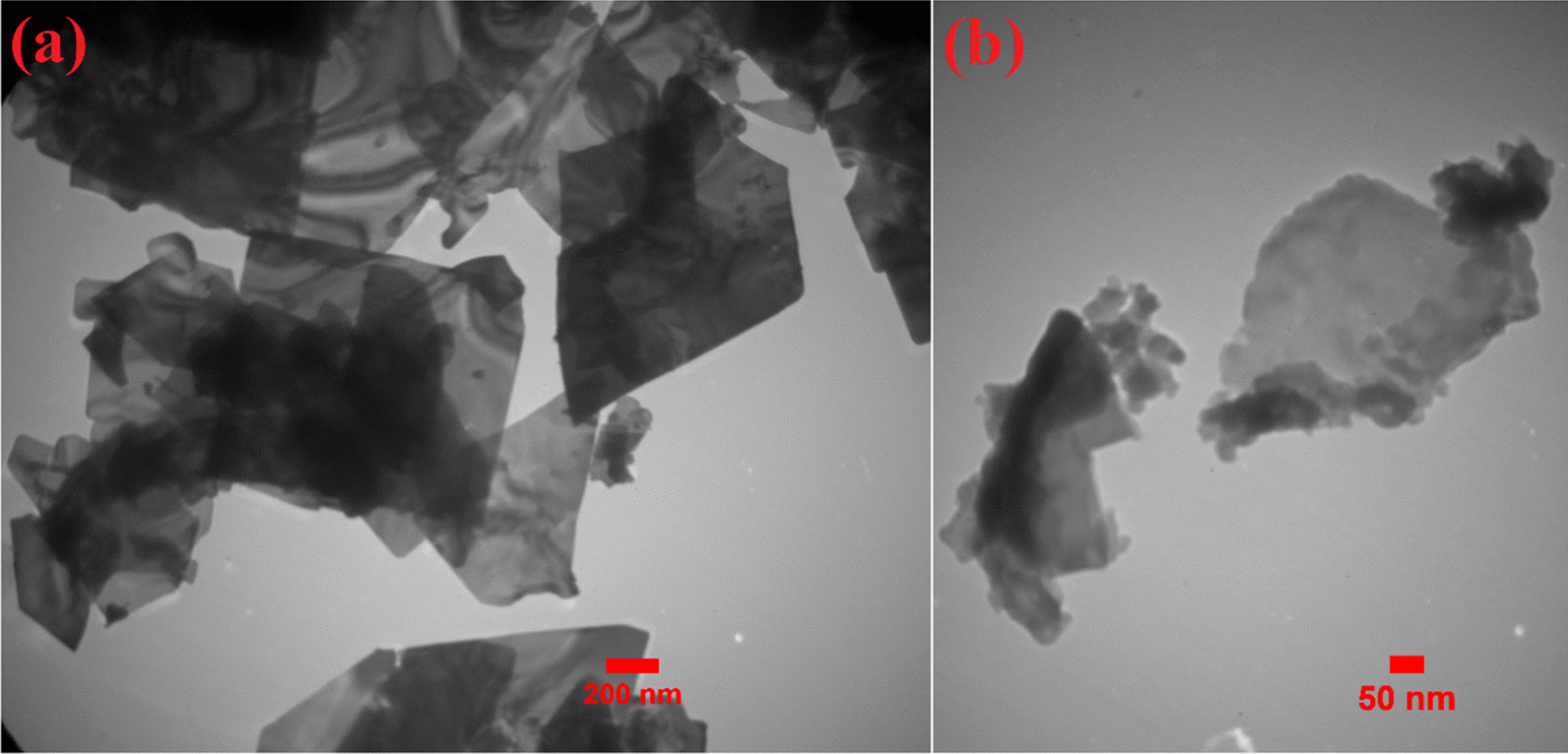
TEM images of Bi_2_O_3_/Bi_2_O_2_CO_3_/(BiO)_4_CO_3_(OH)_2_ nanoflakes with different magnifications, **a** 200 nm and **b** 50 nm

Figure 5a and b shows the TEM image of Bi_2_O_3_/Bi_2_O_2_CO_3_/(BiO)_4_CO_3_(OH)_2_ nanoflakes with different magnifications, which confirms the SEM observation. With the formation of nanostructures with the more effective surface area, the interaction with dye molecules will increase. Therefore, it can be expected that by increasing the photocatalytic reaction sites, photocatalytic activity will improve [66, 67, 68]. Hence, the Bi_2_O_3_/Bi_2_O_2_CO_3_/(BiO)_4_CO_3_(OH)_2_ with fine flake morphology will have more photocatalytic reaction sites and thus better photocatalytic performance than Bi_2_O_3_/Bi/Bi(OH)_3_.

**Fig. 6:**
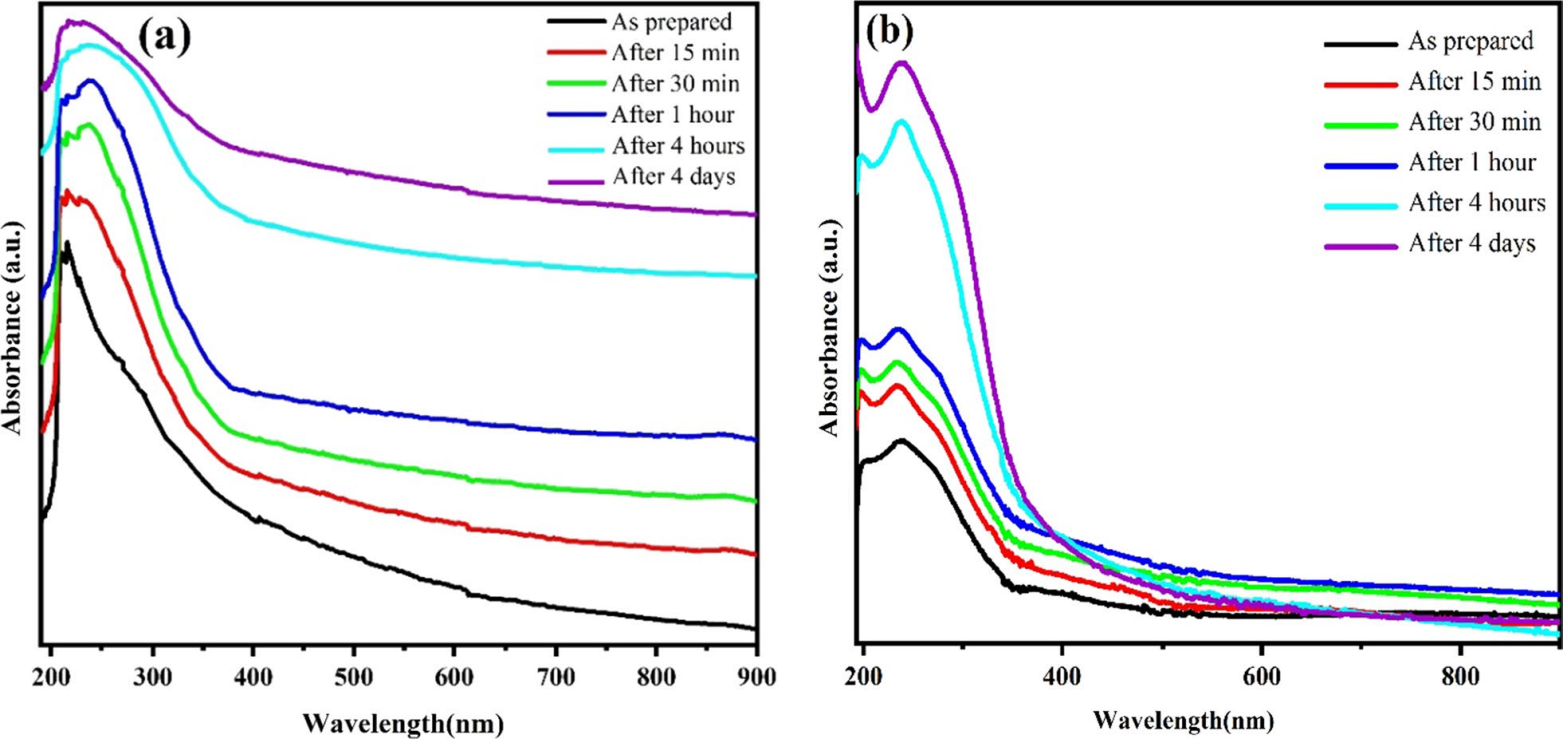
UV–visible optical absorption spectra of synthesized colloidal samples in **a** urea solution, **b** water, over different times

Optical properties, especially the UV–visible spectrum of the samples, are an effective way to evaluate photocatalytic activity via measuring light harvesting efficiency over different ranges of visible spectrum [53]. The optical absorption spectra of colloidal samples over different times immediately after synthesis, 15 min, 30 min, 1 h, 4 h and 4 days are shown in Fig. 6. The spectra are shown at a wavelength between 190 and 900 nm. The intensity and wavelength of the optical absorption peak as well as the form of the spectrum depend on the morphology, size, material type of nanostructures and dielectric function of the surrounding medium [69]. The optical absorption spectra of the samples show an intense absorption at wavelengths between 190 and 400 nm, which is explained by electron transitions from the valance band to the conduction band of Bi_2_O_3_/Bi_2_O_2_CO_3_/(BiO)_4_CO_3_(OH)_2_ and Bi_2_O_3_/Bi/Bi(OH)_3_, which is the feature of semiconductor materials [26, 43, 60]. The intense absorption edge of both samples reveals their proper visible light absorbing ability. In addition, noble metallic nanoparticles reveal an absorption peak in the UV–visible spectrum which is explained by surface plasmon resonance (SPR) [70]. According to XRD results of synthesized sample in water the formation of bismuth phase in fresh samples are probable, hence the observable peak around 200 nm can be attributed to SPR of metallic bismuth phase in both samples [54]. The occurred changes in the absorption spectra can be explained by the disappearing of the bismuth plasmon absorption peak by oxidation of nanostructures. The optical properties and absorption edge of Bi_2_O_3_/Bi_2_O_2_CO_3_/(BiO)_4_CO_3_(OH)_2_ show that synthesized nanoflakes in urea solution can be a good candidate for visible light harvesting and possible photocatalytic activity than Bi_2_O_3_/Bi/Bi(OH)_3_.

**Fig. 7:**
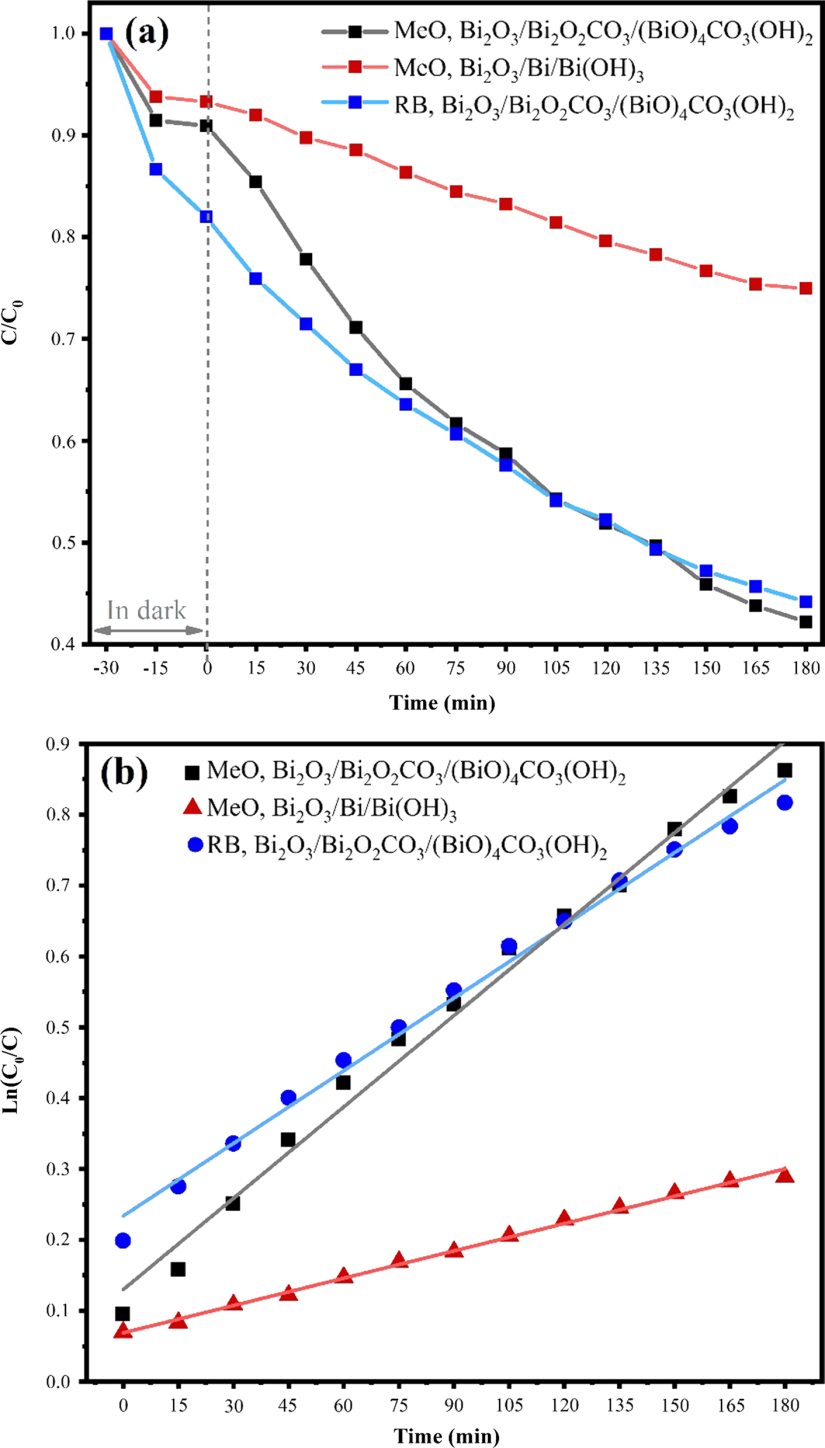
**a** Photocatalytic degradation of dyes and **b** Liner curves of ln(*C*/*C*_0_) versus the irradiation time for the photodegradation reaction kinetics of dyes by samples

The photocatalytic activity of the novel Bi_2_O_3_/Bi_2_O_2_CO_3_/(BiO)_4_CO_3_(OH)_2_ nanoflakes with multi-heterojunctions and Bi_2_O_3_/Bi/Bi(OH)_3_ nanocomposite was evaluated by the degradation of MeO under visible light irradiation for 180 min. In addition, to reveal the capacity of Bi_2_O_3_/Bi_2_O_2_CO_3_/(BiO)_4_CO_3_(OH)_2_ for use as a proper photocatalyst in environmental applications, the photocatalytic performance of Bi_2_O_3_/Bi_2_O_2_CO_3_/(BiO)_4_CO_3_(OH)_2_ investigated for the degradation of RB as well. The optical transmission spectra of MeO solution during the degradation process by Bi_2_O_3_/Bi_2_O_2_CO_3_/(BiO)_4_CO_3_(OH)_2_ nanoflakes exhibited considerable photocatalytic activity compared to that of Bi_2_O_3_/Bi/Bi(OH)_3_. The MeO and RB absorption peaks at 464 and 530 nm were considerably reduced by Bi_2_O_3_/Bi_2_O_2_CO_3_/(BiO)_4_CO_3_(OH)_2_ photocatalyst. The concentration of the dye at different times during degradation can be obtained by Langmuir–Hinshelwood model [71]:

1 $$r = - \frac{{{\text{d}}C\left( t \right)}}{{{\text{d}}t}} = k_{abc} C\left( t \right)$$

where $r$ is the degradation rate of the dye, $C\left( t \right)$ reveals the concentration of dye at time t and $k_{abc}$ is a constant for reaction rate. The degradation kinetics of the dye can be described by the following equation at low concentration of the dye:

2 $$\ln \left( {\frac{{C_{0} }}{C}} \right) = k_{abs} t$$

where *C*_0_ is the initial concentration of dye. The photocatalytic activity and dye degradation kinetics for the samples are presented in Fig. 7, which were obtained at different reaction times during the photodegradation process. Figure 7a illustrates the *C*/*C*_0_ plot versus irradiation time and, as obviously seen, Bi_2_O_3_/Bi_2_O_2_CO_3_/(BiO)_4_CO_3_(OH)_2_ shows a highly efficient degradation rate. Figure 7b shows the degradation reaction kinetics, which were measured from the slopes of the − ln(*C*/*C*_0_) plots versus irradiation time [72]. The calculated ${k}_{abs}$ values for photodegradation of MeO and RB on Bi_2_O_3_/Bi_2_O_2_CO_3_/(BiO)_4_CO_3_(OH)_2_ are 5.2 × 10^–3^ min^−1^ and 4.9 × 10^–3^ min^−1^, respectively, and for photodegradation of MeO on Bi_2_O_3_/Bi/Bi(OH)_3_ is 2.0 × 10^–3^ min^−1^. The promoted photocatalytic performance of Bi_2_O_3_/Bi_2_O_2_CO_3_/(BiO)_4_CO_3_(OH)_2_ can be attributed to its composition, absorption edge, flake morphology and multiphase structure. Based on the obtained results of this research, it can be concluded that the formation of multi-heterojunction can efficiently improve the photocatalytic activity of Bi_2_O_3_/Bi_2_O_2_CO_3_/(BiO)_4_CO_3_(OH)_2_ photocatalyst in comparison with Bi_2_O_3_/Bi/Bi(OH)_3_ photocatalyst. In addition, these results can be compared with other photocatalysts such as nanosheet Bi_2_O_2_CO_3_ and microrod BiOHC_2_O_4_/Bi_2_O_2_CO_3_ with photocatalytic performance for the removal of MeO in 50 mL aqueous solution of 10 ppm dye under 400 W xenon lamp irradiation. Hierarchical BiOHC_2_O_4_/Bi_2_O_2_CO_3_ heterostructure revealed the highest degradation kinetic with 4.7 × 10^–3^ min^−1^ values, which was considerably higher than single BiOHC_2_O_4_ and Bi_2_O_2_CO_3_ [73]. For silver-doped Bi_2_O_3_ photocatalysts with nanosheet morphology in photodegradation of MeO with 20 mg L^−1^ concentration under a 500 W Xenon arc lamp over 180-min irradiation time, the obtained degradation rate was less than 4 × 10^–3^ min^−1^ in the most optimal state [74]. Degussa P25 titania  was used for the removal of rhodamine B with 10 mg in 50 mL water solution under irradiation of 500 W Mercury-Xenon lamp with 6.4 × 10^−4^ min^−1^ degradation rate [8]. In all of these examples, the power of light sources was around 500 W while the photocatalytic results of this research obtained under only 15 W visible LED.

**Fig. 8:**
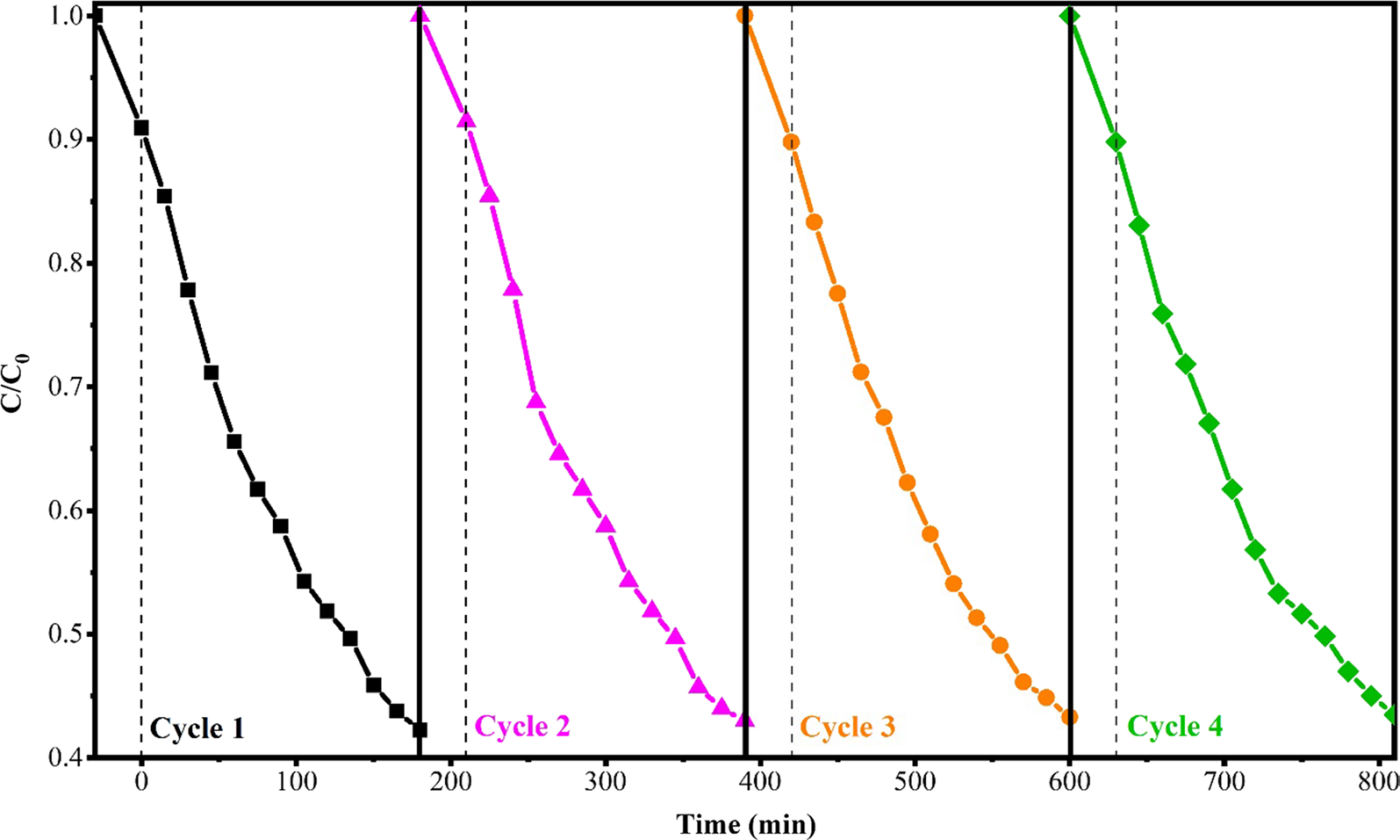
Photocatalytic stability test of Bi_2_O_3_/Bi_2_O_2_CO_3_/(BiO)_4_CO_3_(OH)_2_ photocatalyst

The recyclability and reusability of photocatalysts are important factors in practical applications [75, 76]. The stability of the photocatalytic activity of a photocatalyst can be evaluated through the monitoring of photocatalytic degradation of MeO under light over several cycles. In fact, the stability of a photocatalyst can be proved over every cyclic photocatalytic degradation test [77]. Hence, efficiency should not decline noticeably with each cycle. The degradation efficiency of the recovered Bi_2_O_3_/Bi_2_O_2_CO_3_/(BiO)_4_CO_3_(OH)_2_ photocatalyst after four cycles is shown in Fig. 8. As it is clear, after each cycle the photocatalytic activity of the sample was almost unchanged, which demonstrates high stability of photocatalyst after multiple reuse.

**Fig. 9:**
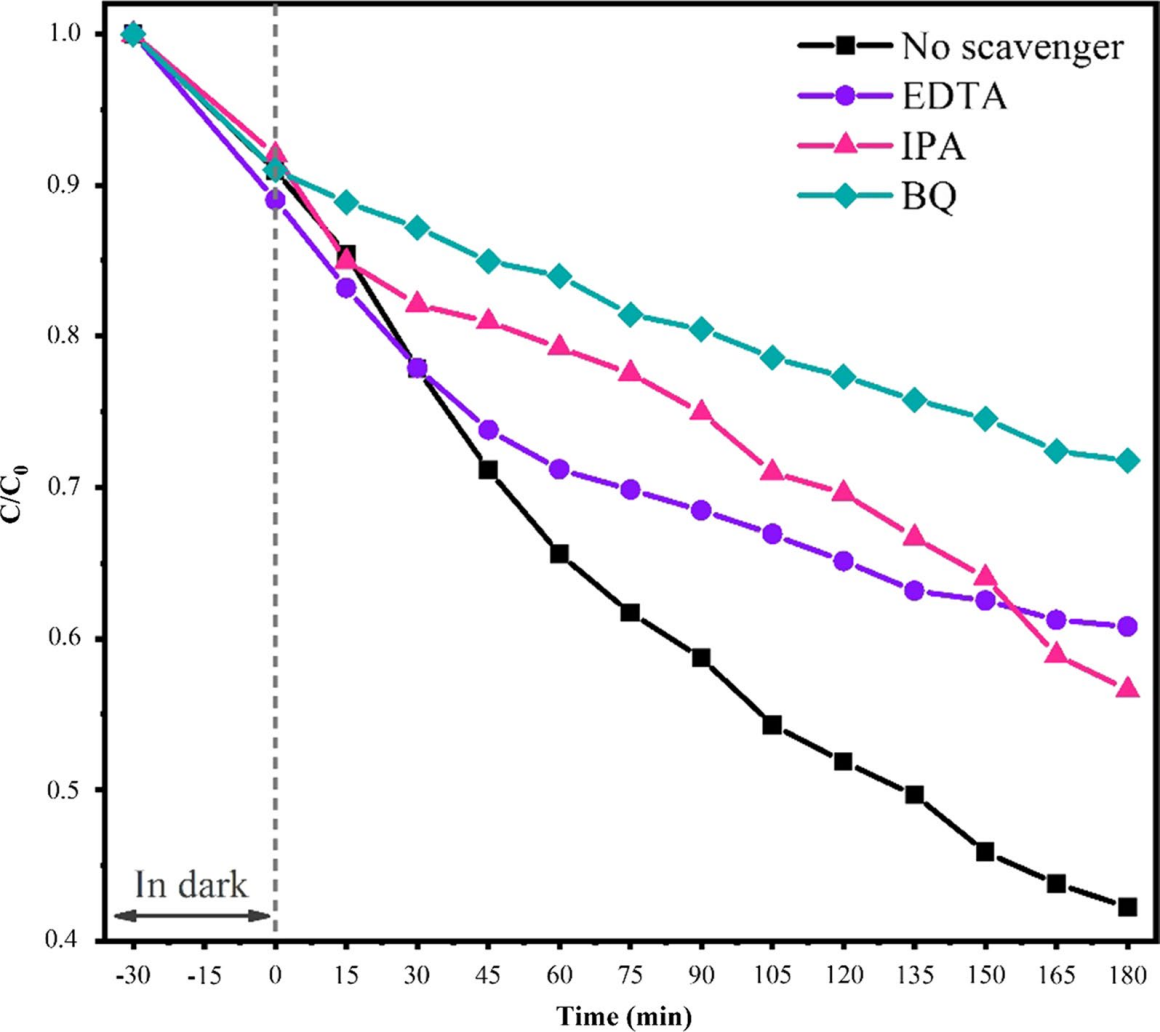
Degradation curves of MeO with different scavengers under the visible light by Bi_2_O_3_/Bi_2_O_2_CO_3_/(BiO)_4_CO_3_(OH)_2_ photocatalyst

The possible photocatalytic degradation mechanism of MeO by Bi_2_O_3_/Bi_2_O_2_CO_3_/(BiO)_4_CO_3_(OH)_2_ under visible light irradiation was investigated through trapping experiments of reactive species in photocatalytic tests by using of different radical scavengers, namely EDTA, IPA and BQ for scavenging the active h^+^, ^•^OH and ^•^O_2_^−^ during the photocatalytic reaction [26, 43]. Based on Fig. 9, degradation values of the MeO decreased in all capturing experiments, which reveals that all the generated reactive species are effective in photodegradation of MeO. However, BQ as a superoxide radical scavenger plays more important role in the MeO degradation. As shown in Fig. 9, by capturing ^•^O_2_^−^ the dye degradation will dwindle more than other h^+^, ^•^OH species.

**Fig. 10:**
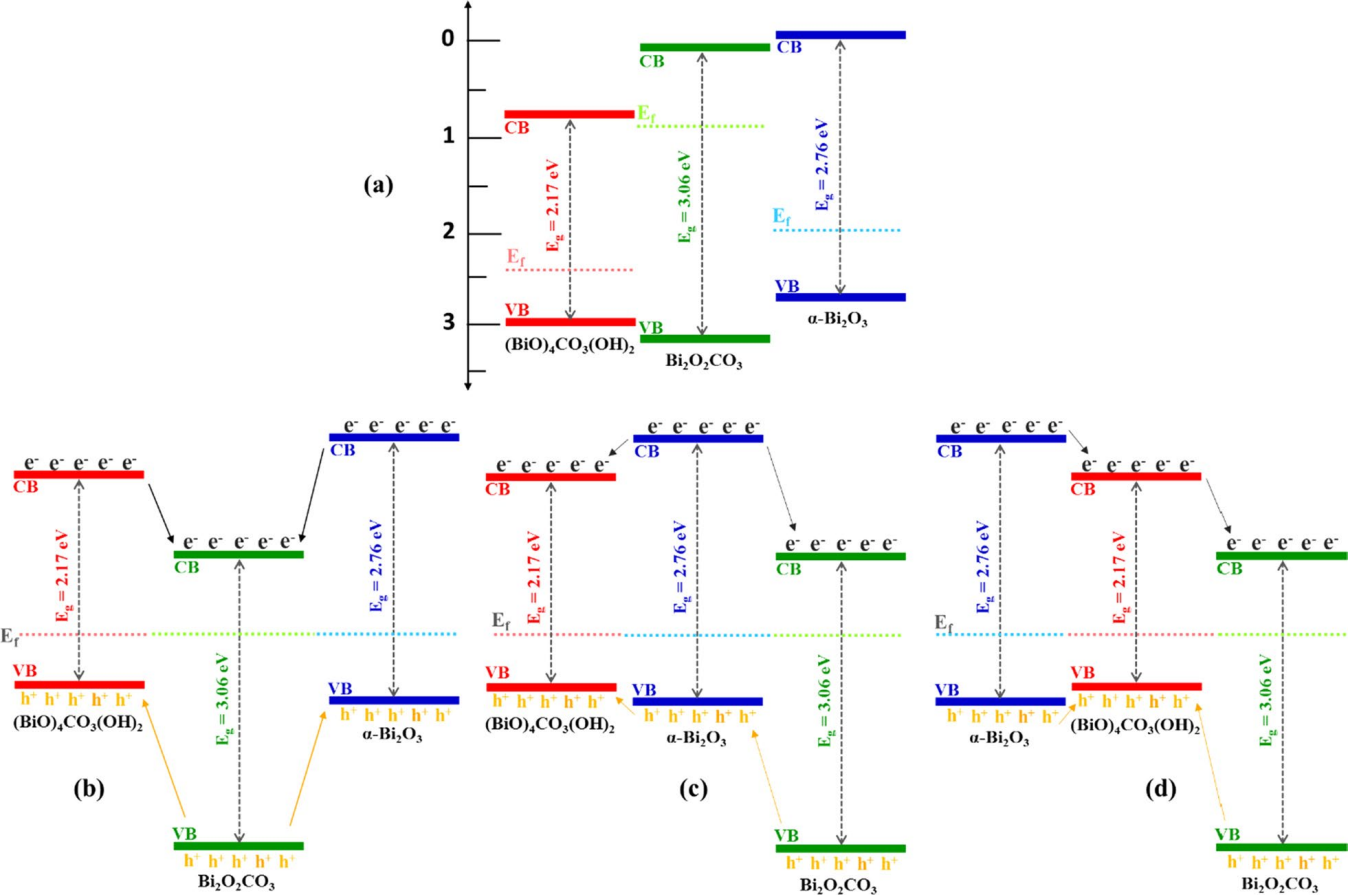
Schematic diagram of three possible photogenerated charge separation for Bi_2_O_3_/Bi_2_O_2_CO_3_/(BiO)_4_CO_3_(OH)_2_, **a** before multi-heterojunctions formation, **b–d** after multi-heterojunctions formation

The photocatalytic performance is deeply depend on the electronic and energy band structure of the semiconductor. Mott–Schottky measurements can be used to better study the energy band structure of a semiconductor. For instance, the band structure of S-scheme BP/BiOBr nano-heterojunction with photocatalytic performance in the degradation of tetracycline and evolution of oxygen, the flat-band potential of BPQDs and CN in CN/rGO@BPQDs high–low junctions system with photocatalytic degradation and H_2_O_2_ production, as well as study of semiconductor type and flat-band potential of S-C_3_N_4_ and PDI-Ala of S-Scheme heterojunction PDI-Ala/S-C_3_N_4_ organic photocatalyst in photodegradation of tetracycline and p-nitrophenol and production of H_2_O_2_ under visible-light irradiation, were measured by the Mott–Schottky method [78, 79, 80]. Although this method has a lot of advantages, it has been used for revealing the band structure of a single component in a multi-component system, while in the present work, the multi-heterojunction Bi_2_O_3_/Bi_2_O_2_CO_3_/(BiO)_4_CO_3_(OH)_2_ nanocomposite has been synthesized during the in situ arc discharge process and there is no chance for the synthesis of single phases with this method. Therefore, the probable band structure of Bi_2_O_3_/Bi_2_O_2_CO_3_/(BiO)_4_CO_3_(OH)_2_ according to the calculated band gap energy level of it and the energy levels of literature are proposed in three possible degradation mechanisms. The energy band structure of Bi_2_O_3_/Bi_2_O_2_CO_3_/(BiO)_4_CO_3_(OH)_2_ multi-heterojunction before and after junction are proposed in Fig. 10 [26, 81, 82]. The Fermi level of α-Bi_2_O_3_ and (BiO)_4_CO_3_(OH)_2_ which are p-type photocatalysts are close to the valence band, and the Fermi level of n-type Bi_2_O_2_CO_3_ is close to the conduction band [26, 83]. By formation of multi-heterojunction, the energy band of α-Bi_2_O_3_, (BiO)_4_CO_3_(OH)_2_ and Bi_2_O_2_CO_3_ will move upward or downward to reach an equilibration state of Fermi level [26]. Under visible light irradiation, the photogenerated electrons in the conduction band of phases will transfer to downer CB, while the holes in the valence band are likely to transfer to the opposite position [82]. In this situation, the recombination of photogenerated carriers in photocatalyst will reduce and thus the formation of p-n-p or p-p-n Bi_2_O_3_/Bi_2_O_2_CO_3_/(BiO)_4_CO_3_(OH)_2_ multi-heterojunction nanoflakes will improve the separation efficiency of the photogenerated electrons and holes which lead better photocatalytic performance.

**Fig. 11:**
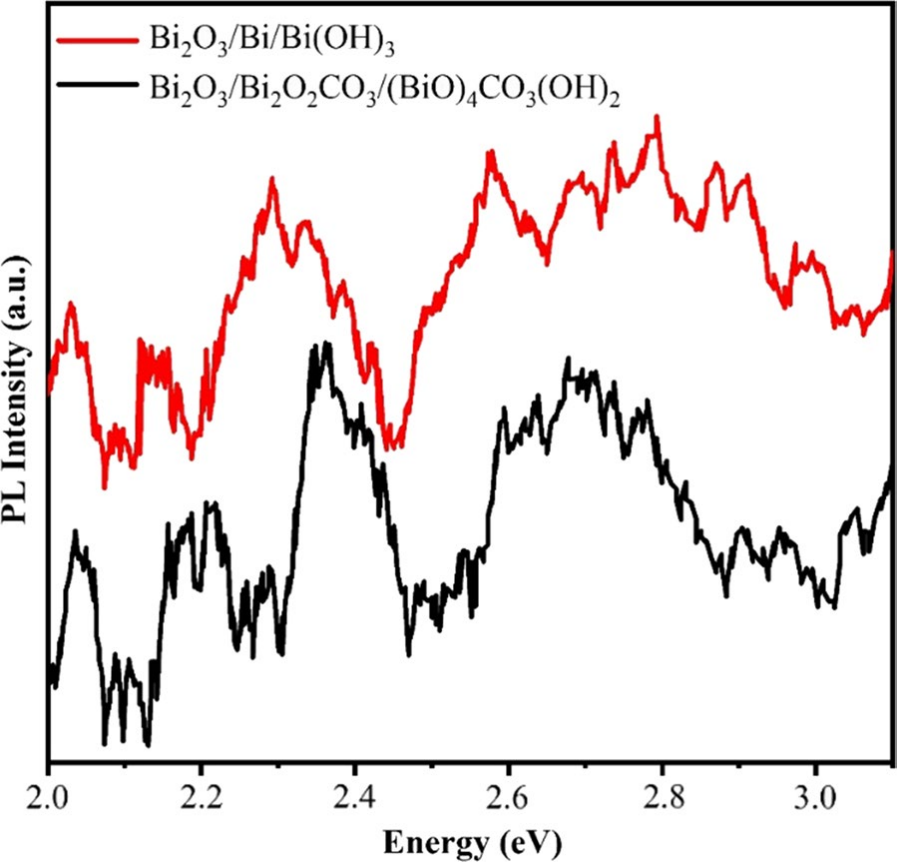
Photoluminescence spectra of synthesized nanostructures by electrical arc discharge in urea solution and water

The PL spectrum of the Bi_2_O_3_/Bi_2_O_2_CO_3_/(BiO)_4_CO_3_(OH)_2_ with the excitation wavelength at 355 nm was measured and compared with the PL spectrum of Bi_2_O_3_/Bi/Bi(OH)_3_ to display the photoactivity enhancement mechanism. Broad emission peaks in the range between 2 and 3 eV are found in the PL spectra of the samples, which is in agreement with the documents [24, 43, 51, 84]. In general, a superior PL intensity shows an upper recombination rate of photogenerated electrons and holes, and thus lower PL intensity expresses a lower recombination rate of photogenerated electrons and holes, which means an improved separation rate of electrons and holes and so better photocatalytic performance [43]. Based on PL spectra shown in Fig. 11, the PL intensities of the multi-heterojunction Bi_2_O_3_/Bi_2_O_2_CO_3_/(BiO)_4_CO_3_(OH)_2_ nanoflakes are all lower than that of the Bi_2_O_3_/Bi/Bi(OH)_3_ at the same condition, which means the efficient separation rate of the carriers in Bi_2_O_3_/Bi_2_O_2_CO_3_/(BiO)_4_CO_3_(OH)_2_. The formation of the multi-heterojunction reduces the recombination rate of photogenerated electrons and holes and then is favorable to improving photocatalytic activity.

## Conclusion

In summary, new multi-heterojunction Bi_2_O_3_/Bi_2_O_2_CO_3_/(BiO)_4_CO_3_(OH)_2_ nanoflakes were prepared by applying a 40 A DC current between two bismuth electrodes through DC electrical arc discharge in urea solution and investigated for using them as a visible light photocatalyst. The photocatalytic performance of Bi_2_O_3_/Bi_2_O_2_CO_3_/(BiO)_4_CO_3_(OH)_2_ is compared with that of Bi_2_O_3_/Bi/Bi(OH)_3_ which is synthesized in the same condition in water. FT-IR, XRD and EDS results revealed the formation of α-Bi_2_O_3_, Bi_2_O_2_CO_3_ and (BiO)_4_CO_3_(OH)_2_ phases. FE-SEM and TEM analysis revealed the fine flake morphology of Bi_2_O_3_/Bi_2_O_2_CO_3_/(BiO)_4_CO_3_(OH)_2_ with a thickness of about 22.0 ± 0.5 nm. The optical absorption spectra of the samplesshow an absorption edge at a wavelength between 190 and 400 nm. The XRD and PL results confirm the formation of multi-heterojunction between α-Bi_2_O_3_, Bi_2_O_2_CO_3_ and (BiO)_4_CO_3_(OH)_2_ phases. In comparison with Bi_2_O_3_/Bi/Bi(OH)_3_, the Bi_2_O_3_/Bi_2_O_2_CO_3_/(BiO)_4_CO_3_(OH)_2_ nanoflakes reveal lower agglomerated morphology, better absorption edge and lower recombination rate and thus much better photocatalytic activity. The results of this research introduce a novel multi-heterojunction compound with considerable multi-applicable photocatalytic performance in environmental applications via an appropriate and cost-effective synthesis method.

## Data Availability

The datasets used and/or analyzed during the current study are available from the corresponding author on reasonable request.

## Abbreviations

DC: Direct current
IEF: Internal electric field
MeO: Methylene orange
FT-IR: Fourier-transform infrared spectroscopy
XRD: X-ray diffraction
FE-SEM: Field emission scanning electron microscopy
EDS: Energy dispersive spectroscopy
TEM: Transmission electron microscopy
PL: Photoluminescence
EDTA: Ethylenediaminetetraacetic acid
IPA: Isopropyl alcohol
BQ: Benzoquinone (BQ)

## References

[1] Qin Z, Su T, Ji H, Guo Z, Chen Y, Lu NL (2018). Photocatalytic nanomaterials for the energy and environmental application. Multifunctional nanocomposites for energy and environmental applications.

[2] Pang H, Wei C, Li X (2015). Microwave-assisted synthesis of NiS_2_ nanostructures for supercapacitors and cocatalytic enhancing photocatalytic H_2_ production. Sci Rep.

[3] Rashid J, Parveen N, Iqbal A (2019). Facile synthesis of g-C_3_N_4_(0.94)/CeO_2_(0.05)/Fe_3_O_4_(0.01) nanosheets for DFT supported visible photocatalysis of 2-Chlorophenol. Sci Rep.

[4] Chen M, Liu P, He J-H (2021). Nanofiber template-induced preparation of ZnO nanocrystal and its application in photocatalysis. Sci Rep.

[5] Li X, Kang B, Dong F (2021). Enhanced photocatalytic degradation and H_2_/H_2_O_2_ production performance of S-pCN/WO_2.72_ S-scheme heterojunction with appropriate surface oxygen vacancies. Nano Energy.

[6] Acharya R, Parida K (2020). A review on TiO_2_/g-C_3_N_4_ visible-light- responsive photocatalysts for sustainable energy generation and environmental remediation. J Environ Chem Eng.

[7] Raizada P, Sharma S, Kumar A (2020). Performance improvement strategies of CuWO_4_ photocatalyst for hydrogen generation and pollutant degradation. J Environ Chem Eng.

[8] Basith MA, Ahsan R, Zarin I, Jalil MA (2018). Enhanced photocatalytic dye degradation and hydrogen production ability of Bi_25_FeO_40_-rGO nanocomposite and mechanism insight. Sci Rep.

[9] Carneiro JO, Teixeira V, Carvalho P et al (2011) Self-cleaning smart nanocoatings. In: Makhlouf ASH, Tiginyanu I (eds) Nanocoatings and ultra-thin films. Elsevier, pp 397–413

[10] Guo Y, Yan B, Deng F (2022). Lattice expansion boosting photocatalytic degradation performance of CuCo_2_S_4_ with an inherent dipole moment. Chin Chem Lett.

[11] Borges ME, Sierra M, Cuevas E (2016). Photocatalysis with solar energy: Sunlight-responsive photocatalyst based on TiO_2_ loaded on a natural material for wastewater treatment. Sol Energy.

[12] Gutierrez-Mata AG, Velazquez-Martínez S, Álvarez-Gallegos A (2017). Recent overview of solar photocatalysis and solar photo-fenton processes for wastewater treatment. Int J Photoenergy.

[13] Mills A, Le Hunte S (1997). An overview of semiconductor photocatalysis. J Photochem Photobiol A.

[14] Hoffmann MR, Martin ST, Wonyong C, Bahnemann DW (1995). Environmental applications of semiconductor photocatalysis. Chem Rev.

[15] Meng X, Zhang Z (2016). Bismuth-based photocatalytic semiconductors: introduction, challenges and possible approaches. J Mol Catal A Chem.

[16] Mehring M (2007). From molecules to bismuth oxide-based materials: potential homo- and heterometallic precursors and model compounds. Coord Chem Rev.

[17] Reverberi AP, Varbanov PS, Vocciante M, Fabiano B (2018). Bismuth oxide-related photocatalysts in green nanotechnology: a critical analysis. Front Chem Sci Eng.

[18] Jansi Rani B, Babu ES, Praveenkumar M (2020). Morphology-dependent photoelectrochemical and photocatalytic performance of γ-Bi_2_O_3_ nanostructures. J Nanosci Nanotechnol.

[19] Khairnar SD, Shrivastava VS (2019). Photocatalytic degradation of chlorpyrifos and methylene blue using α-Bi_2_O_3_ nanoparticles fabricated by sol–gel method. SN Appl Sci.

[20] Schlesinger M, Schulze S, Hietschold M, Mehring M (2013). Metastable β-Bi_2_O_3_ nanoparticles with high photocatalytic activity from polynuclear bismuth oxido clusters. Dalton Trans.

[21] Oudghiri-Hassani H, Rakass S, Al Wadaani FT (2015). Synthesis, characterization and photocatalytic activity of α-Bi_2_O_3_ nanoparticles. J Taibah Univ Sci.

[22] Jalalah M, Faisal M, Bouzid H (2015). Comparative study on photocatalytic performances of crystalline α- and β-Bi_2_O_3_ nanoparticles under visible light. J Ind Eng Chem.

[23] Margha FH, Radwan EK, Badawy MI, Gad-Allah TA (2020). Bi_2_O_3_−BiFeO_3_ glass-ceramic: controllable β-/γ-Bi_2_O_3_ transformation and application as magnetic solar-driven photocatalyst for water decontamination. ACS Omega.

[24] Liu G, Li S, Lu Y (2016). Controllable synthesis of α-Bi_2_O_3_ and γ-Bi_2_O_3_ with high photocatalytic activity by α-Bi_2_O_3_→γ-Bi_2_O_3_→α-Bi_2_O_3_ transformation in a facile precipitation method. J Alloys Compd.

[25] Eberl J, Kisch H (2008). Visible light photo-oxidations in the presence of α-Bi_2_O_3_. Photochem Photobiol Sci.

[26] Xu Z, Wang F, Zhang J (2020). In situ synthesis of p-n (BiO)_4_CO_3_(OH)_2_/Bi_2_O_2_CO_3_ internal polarized heterojunction for improved visible light photocatalytic performance. Mater Res Express.

[27] Riente P, Fianchini M, Llanes P (2021). Shedding light on the nature of the catalytically active species in photocatalytic reactions using Bi_2_O_3_ semiconductor. Nat Commun.

[28] Huang RZ, Wei YY, Gao TF (2021). Structure and electronic properties of δ-Bi_2_O_3_ tuned by vacancy and doping: a first-principles study. Ceram Int.

[29] Huang Y, Wang W, Zhang Q (2016). In situ fabrication of α-Bi_2_O_3_/(BiO)_2_CO_3_ nanoplate heterojunctions with tunable optical property and photocatalytic activity. Sci Rep.

[30] Yakout SM (2020). New efficient sunlight photocatalysts based on Gd, Nb, V and Mn doped alpha-Bi_2_O_3_ phase. J Environ Chem Eng.

[31] Munir S, Rasheed A, Zulfiqar S (2020). Synthesis, characterization and photocatalytic parameters investigation of a new CuFe_2_O_4_/Bi_2_O_3_ nanocomposite. Ceram Int.

[32] Lin Y-C, Peng C-K, Lim S-C (2021). Tailoring the surface oxygen engineering of a carbon-quantum-dot-sensitized ZnO@H-ZnO_1-x_ multijunction toward efficient charge dynamics and photoactivity enhancement. Appl Catal B.

[33] Li X, Luo Q, Han L (2022). Enhanced photocatalytic degradation and H_2_ evolution performance of N-CDs/S-C_3_N_4_ S-scheme heterojunction constructed by π–π conjugate self-assembly. J Mater Sci Technol.

[34] Wang W, Li X, Deng F (2022). Novel organic/inorganic PDI-Urea/BiOBr S-scheme heterojunction for improved photocatalytic antibiotic degradation and H_2_O_2_ production. Chin Chem Lett.

[35] Li X, Liu Q, Deng F (2022). Double-defect-induced polarization enhanced OV-BiOBr/Cu_2−x_S high-low junction for boosted photoelectrochemical hydrogen evolution. Appl Catal B.

[36] Long Y, Li L, Zhou L (2020). Fabrication of the AgI/BiOI/BiPO_4_ multi-heterojunction with high photocatalytic activity. Mater Res Bull.

[37] Yan Q, Xie X, Liu Y (2019). Constructing a new Z-scheme multi-heterojunction photocataslyts Ag–AgI/BiOI-Bi_2_O_3_ with enhanced photocatalytic activity. J Hazard Mater.

[38] Wang K, Qian Z, Guo W (2019). Multi-heterojunction of SnO_2_/Bi_2_O_3_/BiOI nanofibers: facile fabrication with enhanced visible-light photocatalytic performance. Mater Res Bull.

[39] Huang Y, Fan W, Long B (2016). Visible light Bi_2_S_3_/Bi_2_O_3_/Bi_2_O_2_CO_3_ photocatalyst for effective degradation of organic pollutions. Appl Catal B.

[40] Zhou Y, Wang H, Sheng M (2013). Environmentally friendly room temperature synthesis and humidity sensing applications of nanostructured Bi_2_O_2_CO_3_. Sens Actuators B Chem.

[41] Dong F, Lee SC, Wu Z (2011). Rose-like monodisperse bismuth subcarbonate hierarchical hollow microspheres: one-pot template-free fabrication and excellent visible light photocatalytic activity and photochemical stability for NO removal in indoor air. J Hazard Mater.

[42] Dong F, Sun Y, Fu M (2012). Novel in situ N-doped (BiO)_2_CO_3_ hierarchical microspheres self-assembled by nanosheets as efficient and durable visible light driven photocatalyst. Langmuir.

[43] Wang P, Xu L, Ao Y, Wang C (2017). In-situ growth of Au and β-Bi_2_O_3_ nanoparticles on flower-like Bi_2_O_2_CO_3_: a multi-heterojunction photocatalyst with enhanced visible light responsive photocatalytic activity. J Colloid Interface Sci.

[44] Nuñez-Briones A, García-Cerda L, Rodríguez-Hernández J (2018). Synthesis, structural characterization, and photocatalytic activity of Bi-based nanoparticles. Int J Appl Ceram Technol.

[45] Wang W, Cheng H, Huang B (2013). Synthesis of Bi_2_O_2_CO_3_/Bi_2_S_3_ hierarchical microspheres with heterojunctions and their enhanced visible light-driven photocatalytic degradation of dye pollutants. J Colloid Interface Sci.

[46] Zhao Z, Hao Y, Song X, Deng Z (2020). High visible-light rhodamine B degradation activity over two-dimensional Bi_2_O_2_CO_3_/BiOCl heterojunction through the cohesive and efficient electronic transmission channel. J Mater Sci Mater Electron.

[47] Dong F, Ho W-K, Lee SC (2011). Template-free fabrication and growth mechanism of uniform (BiO)_2_CO_3_ hierarchical hollow microspheres with outstanding photocatalytic activities under both UV and visible light irradiation. J Mater Chem.

[48] Sun J, Wang J, Li Z (2015). Assembly and electrochemical properties of novel alkaline rechargeable Ni/Bi battery using Ni(OH)_2_ and (BiO)_4_CO_3_(OH)_2_ microspheres as electrode materials. J Power Sources.

[49] Dong Y, Ma A, Zhang D (2020). Preparation of high-performance α-Bi_2_O_3_ photocatalysts and their photocatalytic activity. Surf Innov.

[50] Dadashi S, Poursalehi R, Delavari HH (2018). Formation, gradual oxidation mechanism and tunable optical properties of Bi/Bi_2_O_3_ nanoparticles prepared by Nd:YAG laser ablation in liquid: dissolved oxygen as genesis of tractable oxidation. Mater Res Bull.

[51] Sun D, Huang L, Li L (2020). Plasma enhanced Bi/Bi_2_O_2_CO_3_ heterojunction photocatalyst via a novel in-situ method. J Colloid Interface Sci.

[52] Yu C, Zhou W, Zhu L (2016). Integrating plasmonic Au nanorods with dendritic like α-Bi_2_O_3_/Bi_2_O_2_CO_3_ heterostructures for superior visible-light-driven photocatalysis. Appl Catal B.

[53] Hashemi E, Poursalehi R, Delavari H (2019). Formation mechanisms, structural and optical properties of Bi/Bi_2_O_3_ One dimensional nanostructures prepared via oriented aggregation of bismuth based nanoparticles synthesized by DC arc discharge in water. Mater Sci Semicond Process.

[54] Hashemi E, Poursalehi R, Delavari H (2022). A comparative study of the effects of phase composition on optical properties and photocatalytic activity of α-/β-/γ-Bi_2_O_3_ multi-heterojunction prepared by submerged DC electrical arc discharge. Mater Technol.

[55] Ziashahabi A, Prato M, Dang Z (2019). The effect of silver oxidation on the photocatalytic activity of Ag/ZnO hybrid plasmonic/metal-oxide nanostructures under visible light and in the dark. Sci Rep.

[56] Farajimotlagh M, Poursalehi R, Aliofkhazraei M (2017). Synthesis mechanisms, optical and structural properties of η-Al_2_O_3_ based nanoparticles prepared by DC arc discharge in environmentally friendly liquids. Ceram Int.

[57] Rahnemai Haghighi N, Poursalehi R (2019). Effect of C/H and C/O ratios on the arc discharge synthesis of titanium carbide nanoparticles in organic liquids. Appl Nanosci.

[58] Hashemi E, Poursalehi R, Delavari H (2022). Shed light on the effect of carrier organic liquids on size, phase composition and optical properties of colloidal bismuth nanoparticles prepared by submerged DC electrical arc discharge. J Mater Res.

[59] Delaportas D, Svarnas P, Alexandrou I (2009). γ-Al_2_O_3_ nanoparticle production by arc-discharge in water: in situ discharge characterization and nanoparticle investigation. J Phys D Appl Phys.

[60] Wang Q, Zhang C, Wu H (2019). Fabrication of β-phase AgI and Bi_2_O_3_ co-decorated Bi_2_O_2_CO_3_ heterojunctions with enhanced photocatalytic performance. J Colloid Interface Sci.

[61] Le VH, Nguyen TH, Nguyen HH (2020). Fabrication and electrochemical behavior investigation of a Pt-loaded reduced graphene oxide composite (Pt@rGO) as a high-performance cathode for dye-sensitized solar cells. Int J Photoenergy.

[62] Bulmahn JC, Tikhonowski G, Popov AA (2020). Laser-ablative synthesis of stable aqueous solutions of elemental bismuth nanoparticles for multimodal theranostic applications. Nanomaterials.

[63] Lamia Bourj Study of CeO_2_–Bi_2_O_3_ system for catalyst and conductivity applications. Autre. Université de Toulon; Université Ibn Zohr (Agadir, Maroc). Faculté des sciences

[64] Taylor P, Sunde S, Lopata VJ (1984) Structure, spectra, and stability of solid bismuth carbonates. Can J Chem 62:2863–2873

[65] Greaves C, Blower SK (1988). Structural relationships between Bi_2_O_2_CO_3_ and β-Bi_2_O_3_. Mater Res Bull.

[66] Zhou L, Wang W, Xu H (2009). Bi_2_O_3_ hierarchical nanostructures: controllable synthesis, growth mechanism, and their application in photocatalysis. Chem Eur J.

[67] Zhang L, Wang W, Zhou L, Xu H (2007). Bi_2_WO_6_ nano- and microstructures: shape control and associated visible-light-driven photocatalytic activities. Small.

[68] Yu J, Xiong J, Cheng B, Liu S (2005). Fabrication and characterization of Ag–TiO_2_ multiphase nanocomposite thin films with enhanced photocatalytic activity. Appl Catal B.

[69] Tilaki RM, Irajizad A, Mahdavi SM (2006). Stability, size and optical properties of silver nanoparticles prepared by laser ablation in different carrier media. Appl Phys A.

[70] Ziashahabi A, Poursalehi R, Naseri N (2018). Shed light on submerged DC arc discharge synthesis of low band gap gray Zn/ZnO nanoparticles: formation and gradual oxidation mechanism. Adv Powder Technol.

[71] Vasanthkumar K, Porkodi K, Selvaganapathi A (2007). Constrain in solving Langmuir-Hinshelwood kinetic expression for the photocatalytic degradation of Auramine O aqueous solutions by ZnO catalyst. Dyes Pigm.

[72] Iyyapushpam S, Nishanthi ST, Pathinettam Padiyan D (2012). Synthesis of room temperature bismuth oxide and its photocatalytic activity. Mater Lett.

[73] Yu C, Liu X, Liu R (2020). Hierarchical BiOHC_2_O_4_/Bi_2_O_2_CO_3_ composite microrods fabricated via insitu anion ion-exchange and their advanced photocatalytic performance. J Alloy Compd.

[74] Li Y, Zhang Z, Zhang Y (2014). Preparation of Ag doped Bi_2_O_3_ nanosheets with highly enhanced visible light photocatalytic performances. Ceram Int.

[75] Zhou L, Cai M, Zhang X (2019). Key role of hydrochar in heterogeneous photocatalytic degradation of sulfamethoxazole using Ag_3_PO_4_-based photocatalysts. RSC Adv.

[76] Su F, Li P, Huang J (2021). Photocatalytic degradation of organic dye and tetracycline by ternary Ag_2_O/AgBr–CeO_2_ photocatalyst under visible-light irradiation. Sci Rep.

[77] Sorathiya K, Mishra B, Kalarikkal A (2016). Enhancement in rate of photocatalysis upon catalyst recycling. Sci Rep.

[78] Li X, Xiong J, Gao X (2020). Novel BP/BiOBr S-scheme nano-heterojunction for enhanced visible-light photocatalytic tetracycline removal and oxygen evolution activity. J Hazard Mater.

[79] Xiong J, Li X, Huang J (2020). CN/rGO@BPQDs high-low junctions with stretching spatial charge separation ability for photocatalytic degradation and H2O2 production. Appl Catal B.

[80] Li X, Liu J, Huang J (2020). All organic S-scheme heterojunction PDI-Ala/S-C_3_N_4_ photocatalyst with enhanced photocatalytic performance. Acta Phys Chim Sin.

[81] Zahid AH, Han Q (2021). A review on the preparation, microstructure, and photocatalytic performance of Bi_2_O_3_ in polymorphs. Nanoscale.

[82] Kong S, An Z, Zhang W (2019). Preparation of hollow flower-like microspherical β-Bi_2_O_3_/BiOCl heterojunction and high photocatalytic property for tetracycline hydrochloride degradation. Nanomaterials.

[83] Liu W, Zhou J, Zhou J (2019). Facile fabrication of multi-walled carbon nanotubes (MWCNTs)/α-Bi_2_O_3_ nanosheets composite with enhanced photocatalytic activity for doxycycline degradation under visible light irradiation. J Mater Sci.

[84] Li H, Hu T, Zhang R (2016). Preparation of solid-state Z-scheme Bi_2_MoO_6_/MO (M Cu, Co 3/4, or Ni) heterojunctions with internal electric field-improved performance in photocatalysis. Appl Catal B.

